# Bridging environmental endocrine-disrupting chemicals and AMI: identification of *MERTK* as a potential diagnostic candidate for AMI via integrated bioinformatics and clinical validation

**DOI:** 10.3389/fcvm.2026.1786276

**Published:** 2026-04-15

**Authors:** Kaiyuan Li, Dianfei Long, Lei Wang, Rui Hao, Yilong Man, Yanfeng Ma

**Affiliations:** 1 Department of Cardiology, The Affiliated Hospital of Xuzhou Medical University, Xuzhou, China; 2 Department of Emergency Medicine, Center Hospital of Shandong First Medical University, Jinan, China; 3 Department of Cardiology, Center Hospital of Shandong First Medical University, Jinan, China

**Keywords:** acute myocardial infarction, endocrine-disrupting chemicals, machine learning, *MERTK*, SMR

## Abstract

**Background:**

Increasing attention has been drawn to the close association between exposure to environmental endocrine-disrupting chemicals (EDCs) and the risk of acute myocardial infarction (AMI). This study aimed to utilize multi-omics approaches to identify EDCs-related diagnostic candidate and elucidate their potential roles in AMI.

**Methods:**

This study integrated toxicological targets of 13 representative EDCs with transcriptomic datasets from AMI cohorts. Machine learning algorithms (LASSO and SVM-RFE) were employed to screen feature genes, and model interpretability was achieved via SHAP analysis. Additionally, Summary-data-based Mendelian Randomization (SMR) and clinical validation in plasma samples were utilized to verify the candidate.

**Results:**

A total of 1,818 potential toxicological targets for EDCs were screened. The machine learning algorithms identified an 8-gene diagnostic signature, which demonstrated superior predictive performance in an external validation set (AUC=0.968). Through intersection analysis, *MERTK* was pinpointed as a critical EDCs-related mediator and exhibited a strong positive correlation with the infiltration of immune cells, such as monocytes and neutrophils. SMR results further confirmed that *MERTK* is a causal risk driver for AMI. Consistently, clinical validation demonstrated that plasma *MERTK* levels were significantly elevated in AMI patients compared to controls.

**Conclusion:**

This study established a multi-omics model linking EDCs exposure to AMI pathogenesis and identified *MERTK* as a key diagnostic candidate potentially mediating EDC-induced AMI. These findings provide novel molecular targets for environmental risk assessment and precision diagnosis.

## 1 Introduction

Acute myocardial infarction (AMI), representing the most severe manifestation of ischemic heart disease, remains a leading cause of global morbidity and mortality, imposing a substantial burden on public health systems (1, 2). Despite significant clinical advancements in managing traditional risk factors such as hypertension, dyslipidemia, and smoking, the incidence of AMI remains persistently high. Increasing evidence suggests that other underappreciated environmental determinants play pivotal roles in disease pathogenesis (3, 4). In this context, environmental exposure, particularly long-term exposure to environmental pollutants, is increasingly recognized as a non-negligible precipitating factor in the pathophysiology of cardiovascular disease (CVD) (5–8).

**Figure 1:**
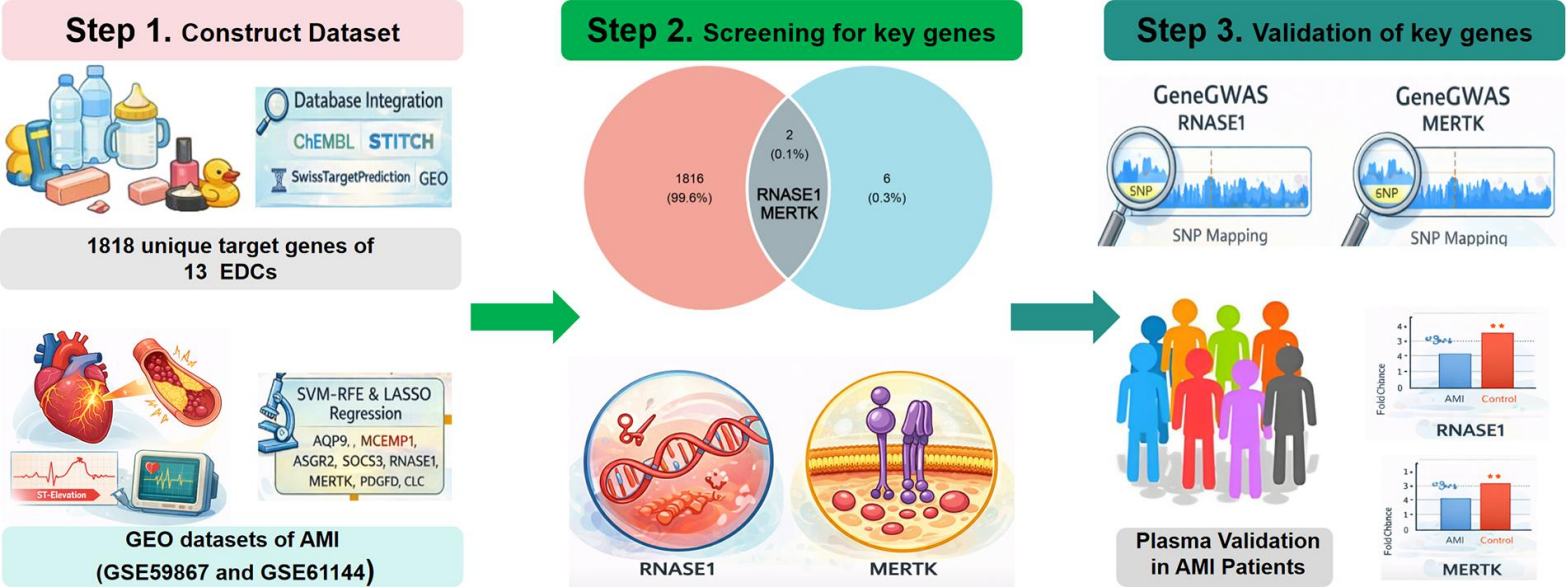
The flow chart of this study.

Among the myriad of environmental risk factors, endocrine-disrupting chemicals (EDCs) have garnered widespread attention due to their ubiquitous environmental presence and potential hazards to human health (9, 10). EDCs are a class of synthetic or naturally occurring compounds capable of interfering with endogenous hormone signaling and metabolic processes, encompassing diverse chemical groups such as bisphenols, phthalates, and polycyclic aromatic hydrocarbons (PAHs) (11, 12). Human exposure to these compounds is extensive, occurring through diet, drinking water, and daily consumer products. Previous toxicological studies have demonstrated that EDCs can induce endothelial dysfunction, oxidative stress, and systemic inflammation, all of which are critical precursors to atherosclerotic plaque instability (13–15). However, while epidemiological evidence suggests a potential link between EDCs exposure and increased cardiovascular risk, the precise molecular bridges connecting environmental toxins to AMI pathogenesis—specifically the key targets mediating alterations in the immune microenvironment—have not yet been fully elucidated. In recent years, advancements in bioinformatics have offered novel opportunities to explore the molecular linkages between environmental exposure and complex diseases. High-throughput databases such as ChEMBL, STITCH, and SwissTargetPrediction enable the systematic prediction of “compound-gene” interactions, while the Gene Expression Omnibus (GEO) provides comprehensive transcriptomic landscapes of disease states. This study aims to construct an integrated multi-omics model bridging environmental EDCs exposure and AMI pathology. By systematically integrating the toxicological targets of 13 representative EDCs with transcriptomic data from AMI patients, we sought to explore potential key molecules located at the intersection of environmental exposure and AMI, thereby providing novel biomarkers and therapeutic targets for environmental risk assessment and precision diagnosis (Figure 1).

## 2 Methods

### 2.1 Selection of environmental EDCs

This study focused on 13 representative environmental EDCs prioritized for public health risk assessment. The panel included bisphenols (BPA, BPS, BPF), organophosphates (chlorpyrifos, diazinon), phthalates (DEHP, DBP), polycyclic aromatic hydrocarbons (PAHs), polychlorinated biphenyls (PCBs), polychlorinated dibenzofurans (PCDFs), per- and polyfluoroalkyl substances (PFOA, PFOS), and 2,3,7,8-tetrachlorodibenzo-p-dioxin (TCDD). These compounds were selected based on their ubiquitous environmental presence, established endocrine-disrupting toxicity, and emerging epidemiological evidence suggesting a link to AMI and cardiovascular susceptibility.

### 2.2 Retrieval and integration of EDCs-target genes

To systematically identify potential human targets for these EDCs, we integrated data from three publicly available chemical biology databases: ChEMBL, STITCH, and SwissTargetPrediction. The canonical SMILES (Simplified Molecular Input Line Entry System) strings for each compound were obtained from the PubChem database and used as queries. To ensure data reliability, strict filtering criteria were applied: ChEMBL Database: Interactions were restricted to Homo sapiens. Only targets with verified experimental bioactivity data (IC50, EC50, Ki, or Kd ≤ 10 μM) were retained to ensure pharmacological relevance. STITCH Database: Predicted chemical–protein interactions were retrieved for Homo sapiens. A minimum confidence score of 0.400 (medium confidence) was applied to exclude low-quality predictions while maintaining comprehensive coverage. SwissTargetPrediction: Targets predicted based on ligand structural similarity were included only if they possessed a probability score >0.1. Post-retrieval, all protein target IDs were standardized to official gene symbols using the UniProtKB and Hugo Gene Nomenclature Committee (HGNC) databases. Non-coding genes and duplicate entries were rigorously removed. Finally, unique targets from all three databases were pooled to construct a comprehensive composite dataset, termed the “Total Union Genes”.

### 2.3 Data integration and batch effect correction

To enhance statistical power and validate the robustness of our findings, raw gene expression profiles from the GSE59867 and GSE61144 datasets were retrieved and harmonized. A rigorous data merging strategy was employed, retaining only genes shared across both platforms. For genes mapped to multiple probes, expression values were averaged to obtain a single expression level per gene. To mitigate non-biological technical variations (batch effects) between datasets, we applied the ComBat algorithm implemented in the sva R package. This method effectively removed technical biases while preserving biological distinctions between the AMI and control groups. The efficacy of batch correction was validated using Principal Component Analysis (PCA), visually confirming the elimination of batch-specific clustering and the homogenization of data distribution.

### 2.4 Differential expression analysis

Following data normalization, differential expression analysis was performed using the limma package. A linear model based on empirical Bayes methods was constructed to identify genes with significant expression changes between AMI patients and healthy controls. Strict selection criteria were applied: a False Discovery Rate (FDR) adjusted *P*-value < 0.05 and an absolute log2 fold change (|logFC|) > 0.585 (representing a > 1.5-fold difference). The overall distribution of differentially expressed genes (DEGs) was visualized using volcano plots, and expression patterns of top significant genes were depicted in hierarchical clustering heatmaps.

### 2.5 Feature selection via machine learning algorithms

To identify the most robust and predictive AMI-related signatures from the identified DEGs, we applied two advanced machine learning strategies. The LASSO regression model was constructed using the glmnet package to perform variable selection and regularization, identifying genes with non-zero coefficients at the optimal lambda (*λ*) determined by cross-validation. Simultaneously, the SVM-RFE algorithm was utilized via the e1071 package to iteratively rank genes and select the optimal subset with the highest discriminatory power. To ensure reliability, only genes consistently selected by both approaches were defined as “Diagnostic Consensus Genes” and prioritized for further investigation.

### 2.6 External validation of diagnostic performance

To verify that the identified markers possess stable diagnostic value across different populations, we conducted an external validation using the GSE34198 dataset as an independent testing cohort. ROC curve analysis was implemented to assess the discriminatory ability of the gene signature. Diagnostic efficacy was quantified by the Area Under the Curve (AUC), along with sensitivity and specificity metrics. High AUC values in this external dataset would corroborate the robustness of the markers as potential non-invasive diagnostic tools for AMI.

### 2.7 Construction of diagnostic model and SHAP analysis

To establish a robust diagnostic classifier, the integrated dataset was randomly stratified into a training set (70%) and an independent testing set (30%) using the caret package. Ten machine learning algorithms were screened using 5-fold repeated cross-validation to ensure stability, and the model achieving the highest AUC was selected as the optimal classifier. To interpret the prediction logic, we employed the SHAP framework and calculated SHAP values via the Permutation SHAP algorithm in the kernelshap package. Using the shapviz package, global feature importance was visualized through beeswarm and bar plots, while non-linear relationships were revealed by dependence plots. Additionally, waterfall and force plots were generated to demonstrate the decision process for individual samples.

### 2.8 Identification of key EDCs-related targets and immune correlation analysis

To pinpoint specific molecular mediators linking environmental exposure to AMI pathology, we performed an intersection analysis between the “Diagnostic Consensus Genes” (Section 2.5) and the “Total Union Genes” (Section 2.2). Genes shared by both datasets were defined as “Key EDCs-Related Diagnostic Genes.” To investigate the potential role of these key genes in regulating the immune response, we analyzed their relationship with immune cell infiltration. The relative abundance of 22 immune cell types was quantified using the CIBERSORT algorithm, focusing specifically on AMI samples to capture disease-specific patterns. The association between gene expression and immune cell fractions was evaluated using Spearman's rank correlation test. Results were visualized using lollipop plots, where dot size represents the correlation coefficient strength and color indicates statistical significance (*P*-value).

### 2.9 Causal inference via summary-data-based mendelian randomization (SMR) analysis

To determine whether the identified key genes play a causal role in AMI, we applied the SMR method. This analysis integrated two large-scale datasets: GWAS summary statistics for AMI from the FinnGen study and cis-eQTL summary data from the eQTLGen consortium. The 1,000 Genomes Project Phase 3 (European population) served as the reference panel to model linkage disequilibrium (LD). The analysis was executed using SMR software (version 1.3.1) with a cis-eQTL scanning window of 500 kb flanking the transcription start site. The SMR test estimated the association strength between gene expression and the AMI phenotype, while the HEIDI (Heterogeneity In Dependent Instruments) test distinguished true causality from pleiotropy (P_HEIDI_ > 0.05). Genes were identified as potential causal drivers if they showed a significant SMR association (P_SMR_ < 0.05) and lack of heterogeneity. Visualizations included SMR effect plots (linear relationship between eQTL and GWAS effect sizes) and locus plots (genomic colocalization of signals).

### 2.10 Validation in clinical plasma samples

To verify the key genes, we recruited AMI patients (*n* = 22) and healthy controls (*n* = 15) at Center Hospital of Shandong First Medical University (Jinan Central Hospital). The study was conducted in accordance with the Declaration of Helsinki and was approved by the Medical Ethics Committee of Jinan Central Hospital (Approval No. 2021-206-02). All participants provided informed consent. Inclusion criteria for AMI were: (1) typical chest pain; (2) ischemic ECG changes; and (3) elevated cardiac biomarkers (cTnI or cTnT). Controls were healthy volunteers. Participants with cancer, severe infections, or autoimmune diseases were excluded.

### 2.11 Sample processing and qRT-PCR

Peripheral blood was collected in EDTA tubes and centrifuged at 3,000 rpm for 10 min to isolate plasma, which was stored at −80 °C. Total RNA was isolated using the Serum/Plasma Total RNA Extraction Kit (Tiangen Biotech, Beijing, China). cDNA was synthesized using the PrimeScript RT reagent Kit, and qRT-PCR was performed using the SYBR Green system with GAPDH as the internal control. Relative gene expression was calculated using the 2^−ΔΔCt^ method. Primer sequences are listed in Table 1.

**Table 1:** Primer sequences.

Gene name	Primer sequence (5′ to 3′)
hsa-MERTK-Forward primer	CCGCCCCACCTTTTCAGTAT
hsa-MERTK-Reverse primer	GAGCTCTCCAGCAACTGTGT
hsa-RNASE1-Forward primer	ATAGGAACTGACAGGATTTTAGGTC
hsa-RNASE1-Reverse primer	CAGAAGGAGCCGGACAAGAG
hsa-GAPDH-Forward primer	CCGTTGAATTTGCCGTGA
hsa-GAPDH-Reverse primer	TGATGACCCTTTTGGCTCCC

## 3 Results

### 3.1 Identification of 1,818 unique target genes for environmental EDCs

**Figure 2:**
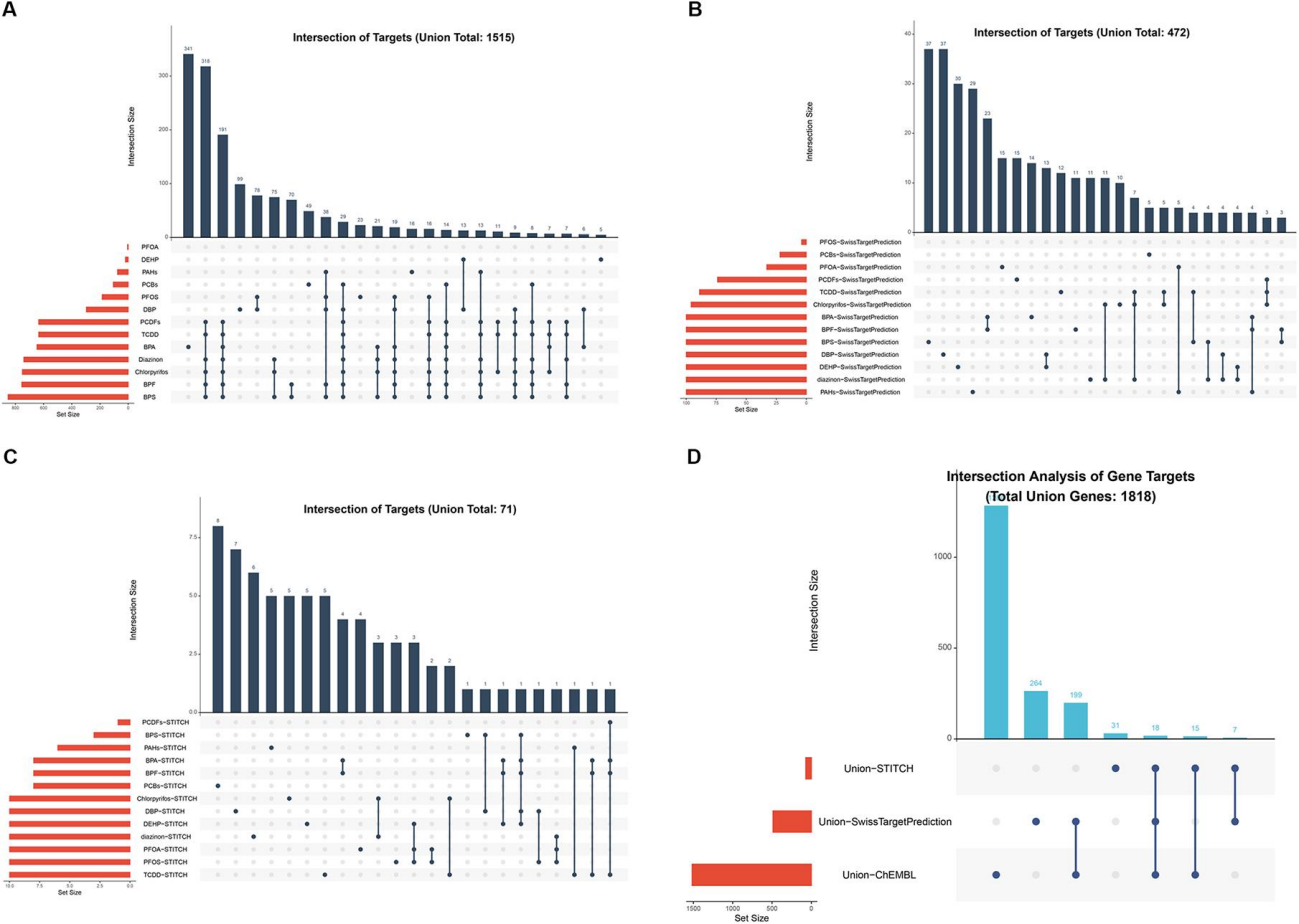
Identification and integration of potential target genes for 13 environmental EDCs. **(A–C)** Venn diagrams displaying the retrieval of target genes associated with the 13 representative EDCs from three independent chemical biology databases: ChEMBL **(A)**, STITCH **(B)**, and SwissTargetPrediction **(C)**. **(D)** Venn diagram illustrating the integration process, resulting in a final pool of 1,818 unique “Total Union Genes”.

Target genes for the 13 EDCs were retrieved from the ChEMBL (*n* = 1,516), STITCH (*n* = 71), and SwissTargetPrediction (*n* = 488) databases (Figures 2A–C). Following data integration and de-duplication, the total Union Genes of 1,818 unique target genes was obtained (Figure 2D), serving as the comprehensive resource for subsequent analyses.

### 3.2 Differential expression analysis produced 49 robust DEGs

**Figure 3:**
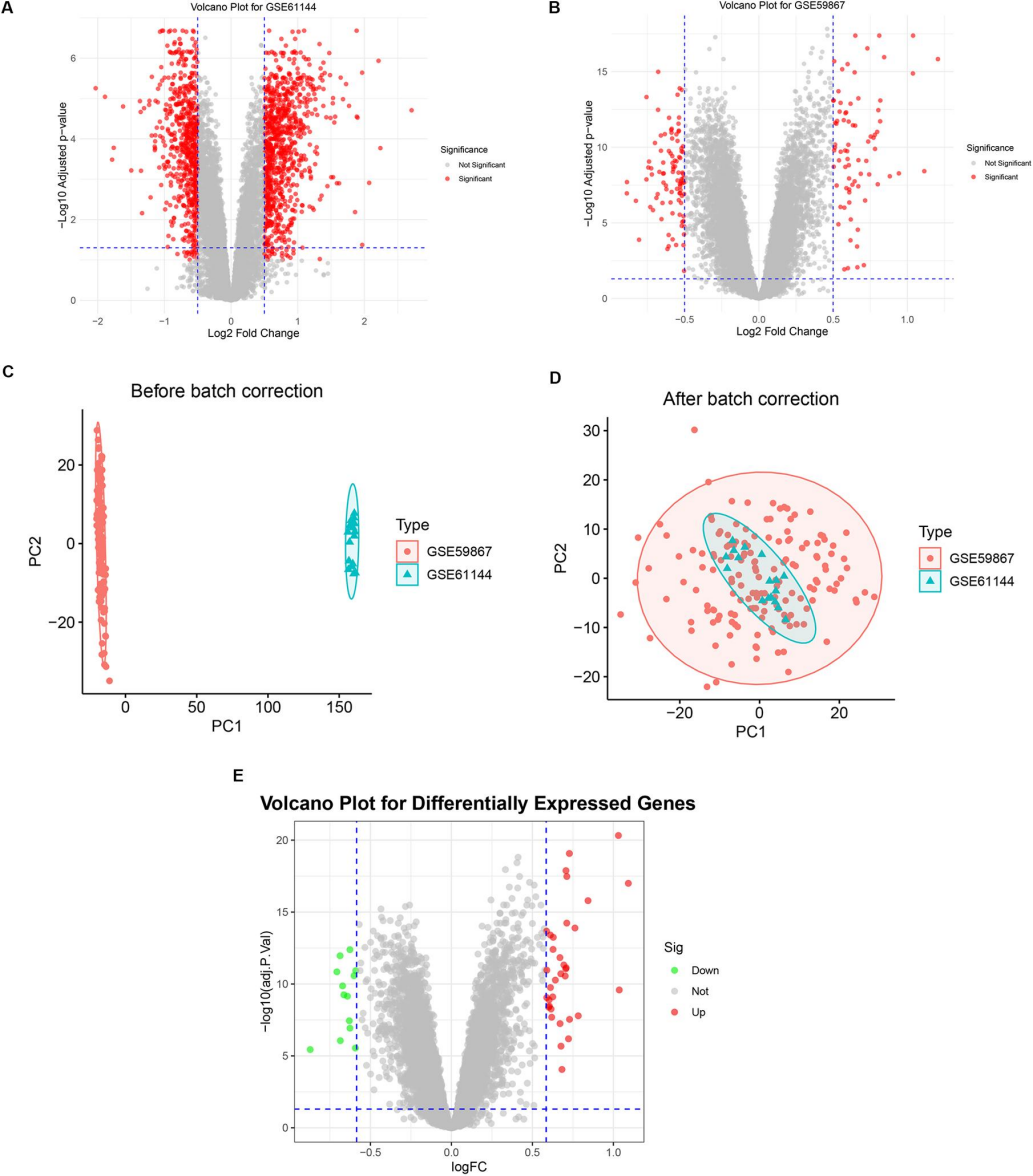
Identification of robust DEGs in AMI through dataset integration and batch effect correction. **(A,B)** Volcano plots showing DEGs identified in two independent datasets: GSE59867 **(A)** and GSE61144 **(B)**. **(C,D)** PCA plots visualizing the sample distribution before **(C)** and after **(D)** applying the ComBat batch correction algorithm. **(E)** Volcano plot displaying the 49 robust DEGs identified from the integrated AMI cohort.

Differential expression analysis was initially performed on two independent datasets, GSE59867 and GSE61144, identifying 174 and 1,627 DEGs, respectively (Figures 3A,B). To enhance robustness and mitigate batch effects, the datasets were integrated. PCA confirmed effective data merging and elimination of batch-specific clustering (Figures 3C,D). Subsequent differential expression analysis on this integrated cohort yielded a total of 49 robust DEGs (Figure 3E).

### 3.3 8 genes were selected by machine learning

**Figure 4:**
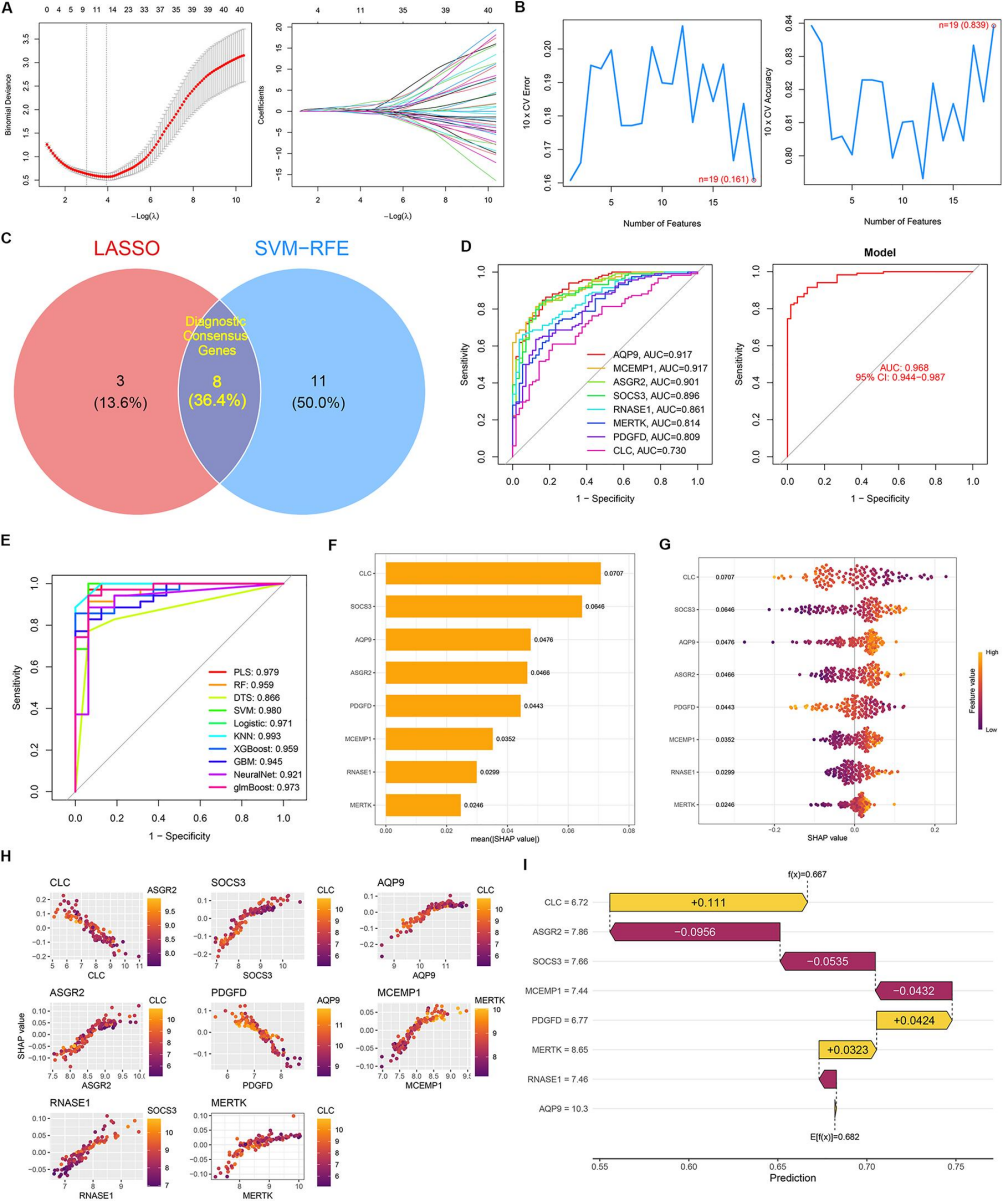
Identification of diagnostic genes and construction of an interpretable machine learning model. **(A)** LASSO regression identified 11 candidate genes. **(B)** SVM-RFE selected 19 feature genes. **(C)** Intersection analysis identified 8 diagnostic consensus genes: AQP9, MCEMP1, ASGR2, SOCS3, RNASE1, MERTK, PDGFD, and CLC. **(D)** ROC analysis showed good diagnostic performance of the 8-gene signature in the external validation dataset (AUC = 0.968). **(E)** Comparison of ten machine learning algorithms identified KNN as the optimal model (AUC = 0.993). **(F)** SHAP summary plot showed the relative importance of each feature. **(G)** Global interpretability analysis illustrated the directional effects of the features on model prediction. **(H)** SHAP dependence plots demonstrated the relationships between gene expression and risk contribution. **(I)** SHAP waterfall plot showed the contribution of each feature to the prediction for a representative AMI sample.

To identify potential diagnostic candidate from the 49 DEGs, we employed two machine learning algorithms. LASSO regression analysis identified 11 candidate genes, including *AQP9*, *S100A9*, and *MCEMP1* (Figure 4A). Concurrently, the SVM-RFE algorithm screened 19 feature genes, such as *RNASE1*, *AQP9*, and *ASGR2* (Figure 4B). By intersecting the candidates identified by both algorithms, we obtained a robust set of 8 diagnostic consensus genes (*AQP9*, *MCEMP1*, *ASGR2*, *SOCS3*, *RNASE1*, *MERTK*, *PDGFD*, *CLC*) (Figure 4C). To verify the diagnostic performance of this gene signature, we performed ROC analysis using an external AMI dataset. The results showed an AUC of 0.968, demonstrating the excellent diagnostic capability and robustness of these 8 genes for distinguishing AMI samples (Figure 4D).

### 3.4 Robustness and interpretability of the diagnostic model

To validate the diagnostic efficacy of the identified gene signature, we compared ten machine learning algorithms. The K-Nearest Neighbors (KNN) model demonstrated superior performance (AUC = 0.993) (Figure 4E). Subsequent SHAP analysis elucidated the model's decision-making process, identifying CLC as the most influential feature, followed by *SOCS3* and *AQP9* (Figure 4F). Global interpretability analysis revealed distinct directional impacts: elevated expression of *CLC* and *PDGFD* functioned as protective factors associated with lower risk scores, whereas *SOCS3*, *AQP9*, *ASGR2*, *MCEMP1*, *RNASE1*, and *MERTK* acted as risk factors (Figure 4G). SHAP dependence plots further revealed clear nonlinear relationships between gene expression levels and model-derived risk contributions for several key genes (Figure 4H). These findings suggest that the influence of transcriptomic perturbations on AMI risk is not purely linear, but may involve threshold effects or context-dependent amplification, thereby supporting the biological complexity of EDCs-related molecular responses. Finally, individual-level visualization via waterfall plots exemplified the model's capability to integrate these complex feature interactions for personalized diagnostic predictions (Figure 4I).

### 3.5 *RNASE1* and *MERTK* as potential mediators of EDCs-induced immune alteration

**Figure 5:**
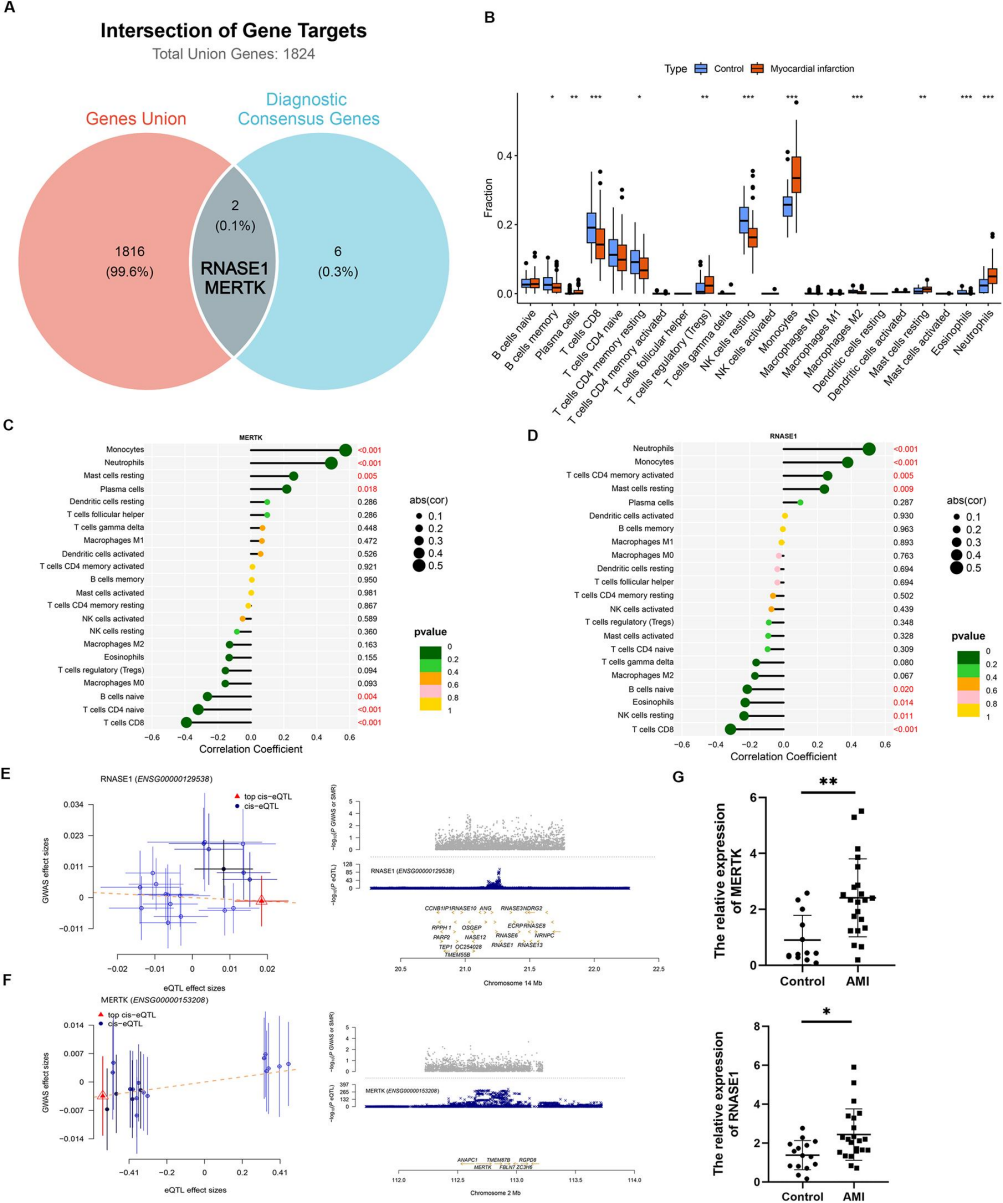
Identification of MERTK as a key EDC-related mediator: immune correlation, causal inference, and clinical validation. **(A)** Venn diagram showing the intersection between the 8 diagnostic consensus genes and the 1,818 EDC target genes, identifying MERTK and RNASE1 as key EDC-related targets. **(B)** Heatmap showing the correlations between the 8 diagnostic genes and 22 immune cell types. **(C,D)** Lollipop plots illustrating the specific correlations of MERTK **(C)** and RNASE1 **(D)** with immune cell infiltration. **(E)** SMR effect plot for RNASE1, showing a negative slope and suggesting that genetically predicted higher expression is associated with reduced AMI risk. **(F)** SMR effect plot for MERTK, showing a positive slope and supporting a potential causal association between elevated MERTK expression and increased AMI risk. **(G)** Validation of relative gene expression in plasma from a clinical cohort (AMI patients, *n* = 22; healthy controls, *n* = 15) by qRT-PCR.

The intersection of the 8 diagnostic consensus genes with the Total Union Genes pinpointed *RNASE1* and *MERTK* as critical genes potentially regulated by environmental EDCs (Figure 5A). Given the pivotal role of inflammation in AMI, we analyzed the correlation between these genes and immune cell infiltration (Figure 5B). Results indicated that *MERTK* was positively correlated with pro-inflammatory cells such as Monocytes and Neutrophils, as well as resting Mast cells and Plasma cells, while inversely correlated with adaptive immune cells like naive B cells, naive CD4+ T cells, and CD8+ T cells (Figure 5C). A consistent pattern was observed for *RNASE1*, which positively correlated with Neutrophils, Monocytes, activated memory CD4+ T cells, and resting Mast cells, while negatively correlating with naive B cells, Eosinophils, resting NK cells, and CD8+ T cells (Figure 5D).

### 3.6 *MERTK* is causally associated with increased AMI risk

SMR analysis was conducted to validate the causal effects of the candidate genes. For *RNASE1*, the plot of GWAS vs. eQTL effect sizes showed a negative slope, indicating that higher genetically predicted expression of *RNASE1* causally correlates with reduced AMI risk (Figure 5E). Conversely, *MERTK* exhibited a positive slope, confirming that elevated expression levels are causally linked to a higher risk of AMI (Figure 5F). These results substantiate the potential of *MERTK* as a risk driver and *RNASE1* as a protective factor in AMI pathogenesis.

### 3.7 MERTK and RNASE1 levels are elevated in AMI patients

To validate the clinical relevance of the identified genes, we analyzed plasma samples from a cohort consisting of 22 AMI patients and 15 healthy controls. The baseline characteristics of the study population are summarized in Table 2. Statistical analysis revealed significant differences between the two groups in terms of Age, E Peak, Total Cholesterol, and High-Density Lipoprotein. Subsequently, we detected the expression levels of the candidate genes in plasma. The results demonstrated that the levels of both *MERTK* and *RNASE1* were significantly elevated in the AMI group compared to the control group (Figure 5G).

**Table 2:** Characteristics of patients.

Indexs	Control (*n* = 15)	AMI (*n* = 22)	*P*-value
Age (y)	48.8 ± 15.8	66.0 ± 15.1	<0.001
LVEF (%)		59.8 ± 9.2	
E Peak	123.07 ± 12.73	137.5 ± 15.9	<0.001
A peak	73.93 ± 12.72	77.6 ± 15.4	0.303
Heart rate (beats/min)	74.87 ± 13.56	80.5 ± 23.3	0.254
TG (mmol/L)	1.47 ± 0.47	1.45 ± 0.76	0.904
TC (mmol/L)	3.09 ± 0.61	4.40 ± 1.11	<0.001
LDL (mmol/L)	2.57 ± 0.45	2.39 ± 0.93	0.333
HDL (mmol/L)	2.15 ± 0.71	1.13 ± 0.40	<0.001

## 4 Discussion

EDCs is considered a potential environmental driver for the rising incidence of cardiovascular diseases; however, the molecular networks through which EDCs disrupt cardiovascular homeostasis remain to be elucidated (16, 17). By integrating toxicological targets with transcriptomic features, this study constructed a diagnostic model comprising eight genes, including *MERTK* and *RNASE1*. The high discriminatory power of this model in an external dataset (AUC = 0.968) suggests that environmental toxins may play a role in the pathogenesis of AMI by perturbing the expression of these key nodes. SHAP-based interpretability analysis further revealed the non-linear contribution of these genes to disease risk. Notably, *CLC* (Charcot-Leyden Crystal Galectin) was identified as the most significant protective feature, with its high expression associated with reduced AMI risk. Previous studies indicate that *CLC* is primarily secreted by eosinophils and may participate in immune regulation and tissue repair (18, 19). Conversely, the high weight assigned to inflammation-related genes, such as *SOCS3* and *AQP9*, may reflect a chronic inflammatory state triggered by EDCs exposure (20, 21). These complex feature combinations unveil the multifaceted nature of AMI pathogenesis and underscore the multidimensional interaction between environmental stress and host gene networks, thereby providing a novel perspective for understanding AMI heterogeneity from the standpoint of environmental medicine.

By mapping the diagnostic features to the EDCs target library, *MERTK* and *RNASE1* were pinpointed as potential molecular effectors of environmental exposure. Although they differ in their classical biological functions, our immune infiltration analysis demonstrated significant synergy within the AMI microenvironment: both genes showed positive correlations with innate immune cells, such as monocytes and neutrophils, and negative correlations with adaptive immune cells, such as CD8+ T cells. This phenomenon implies that EDCs exposure may induce an “innate immune-dominant” microenvironment, facilitating the recruitment of myeloid cells to the injured myocardium. Specifically, *MERTK*, as a member of the TAM receptor family, plays a pivotal role in macrophage-mediated efferocytosis (22, 23). EDCs exposure may mimic endogenous ligands or interfere with receptor signaling, leading to the aberrant upregulation of *MERTK*. While this may initially aid in the clearance of damaged cardiomyocytes, sustained overexpression could be linked to impaired lipid phagocytosis and foam cell formation within plaques, a hypothesis that warrants further experimental verification (24, 25). Concurrently, *RNASE1* is responsible for clearing extracellular RNA to preserve vascular barrier function, and its co-expression with inflammatory cells likely reflects the complex response of the microenvironment to vascular injury (26, 27). Distinguishing whether observed gene expression changes are “initiating factors” or “adaptive consequences” is crucial for understanding pathological mechanisms. Based on our comprehensive analysis, we preliminarily identify *MERTK* as a key pathogenic molecule linking EDCs exposure to AMI. The SMR analysis results revealed a potential causal association between genetic susceptibility to *MERTK* and increased AMI risk; this aligns with the high expression trends observed in clinical samples, strongly supporting its role as a risk driver. In contrast, RNASE1 presents a phenomenon of “genotype-phenotype dissociation”: SMR indicates that its genetically predicted high expression is a protective factor, whereas its actual levels are significantly elevated in the plasma of AMI patients. This seemingly paradoxical phenomenon may reflect a compensatory protective response of the host. RNASE1 is a vascular protective ribonuclease that degrades extracellular RNA (eRNA), a danger-associated molecule released during tissue injury and ischemia (28, 29). Excessive eRNA has been reported to promote endothelial activation, vascular permeability, leukocyte recruitment, and thrombo-inflammatory responses (27). Therefore, increased RNASE1 expression in AMI may represent an adaptive mechanism aimed at limiting eRNA-mediated vascular injury and inflammatory amplification. This interpretation is supported by previous studies showing that *RNASE1* contributes to endothelial homeostasis and may counteract vascular inflammation under acute injury conditions (26, 28). We further hypothesize that during the acute phase of AMI, ischemia-induced eRNA release triggers compensatory upregulation of *RNASE1*; however, under sustained inflammatory or toxic stress, including EDCs exposure, this protective response may be insufficient, delayed, or overwhelmed. This hypothesis is testable in future studies by dynamically monitoring circulating eRNA and *RNASE1* levels in animal models subjected to EDCs exposure followed by AMI induction, and by assessing whether modulation of *RNASE1* alters inflammatory injury and cardiac outcomes. In summary, while both genes are potentially regulated by environmental factors, *MERTK* is more likely to act as a “driver” mediating EDCs pathogenicity, whereas *RNASE1* is more involved in the subsequent “response” process, though this requires further experimental validation.

This study also has limitation. First, this study is based on retrospective data mining; although external validation was performed, the direct link between gene signatures and environmental exposure remains a statistical inference, lacking direct quantification of EDCs burden in individual patients. Second, although multiple machine learning algorithms were compared and SHAP analysis was applied to improve model interpretability, the optimal classifier still retains certain “black-box” characteristics relative to traditional regression-based models. The 8-gene signature achieved a high AUC in the external validation dataset, the possibility of model optimism or partial overfitting cannot be completely excluded, particularly given the relatively limited sample size and the complexity of feature selection. Therefore, its biological transparency and clinical generalizability should be further validated in larger, multicenter, prospective cohorts. Third, while SMR analysis provides supportive evidence for causality, it relies on the validity of genetic instruments and specific model assumptions. Although the HEIDI test helped exclude linkage-driven associations and some forms of pleiotropy, it cannot completely rule out all potential horizontal pleiotropy that may influence the observed associations. In addition, the SMR analysis was based primarily on summary statistics from European-ancestry populations (eQTLGen and FinnGen). Given potential cross-ancestry differences in allele frequencies, linkage disequilibrium structure, and genetic architecture, the generalizability of these findings to non-European populations, particularly East Asian populations, should be interpreted with caution and requires further validation. Moreover, significant between-group differences in age, total cholesterol, and high-density lipoprotein levels were observed. Because the present qRT-PCR analysis was based primarily on unadjusted group comparisons, the potential influence of these clinical variables cannot be fully excluded. Future studies with larger sample sizes, better-matched controls, and multivariable adjustment will be necessary to determine whether *MERTK* and *RNASE1* provide independent diagnostic value beyond conventional clinical factors. Finally, the specific regulatory mechanisms of *MERTK* and *RNASE1* under distinct EDCs exposures (e.g., receptor signaling perturbation, epigenetic modification, or transcriptional regulation) have not yet been confirmed in *in vivo* or *in vitro* experimental models. Future studies should combine prospective exposure assessment, multicenter validation, and mechanistic toxicological experiments to further clarify the “environment-gene” interaction network in AMI.

## 5 Conclusion

This study established a multi-omics framework linking EDCs exposure to AMI pathogenesis and identified *MERTK* as a key diagnostic candidate with potential relevance to EDCs-associated AMI. These findings provide novel clues for environmental cardiovascular risk assessment and precision medicine. Future studies should focus on validating these findings in larger, multi-ethnic, and prospective cohorts, while integrating direct exposure assessment and mechanistic experiments to clarify how EDC-related molecular perturbations contribute to AMI susceptibility and progression. Importantly, from the perspective of environmental medicine, these findings may contribute to early risk stratification and more targeted prevention strategies for cardiovascular high-risk populations with potential environmental exposure burdens.

## Data Availability

The original contributions presented in the study are included in the article/Supplementary Material, further inquiries can be directed to the corresponding authors.
